# Ascorbate degradation: pathways, products, and possibilities

**DOI:** 10.1093/jxb/erae048

**Published:** 2024-02-13

**Authors:** Christopher M Ford, Crystal Sweetman, Stephen C Fry

**Affiliations:** School of Agriculture, Food and Wine and Waite Research Institute, The University of Adelaide, 5005, Australia; College of Science and Engineering, Flinders University, GPO Box 2100, Adelaide 5001, South Australia; The Edinburgh Cell Wall Group, Institute of Molecular Plant Sciences, The University of Edinburgh, The King’s Buildings, Max Born Crescent, Edinburgh EH9 3BF, UK; The James Hutton Institute, UK

**Keywords:** Apoplastic, ascorbate, oxalate, protoplasmic, raphides, tartrate, threonate

## Abstract

A role for l-ascorbate as the precursor of several plant compounds adds to its already broad metabolic utility. There are many examples of plant species in which oxalate and l-threonate are formed from l-ascorbate breakdown, and a number of roles have been proposed for this: structural, physiological, and biochemical. On the other hand, the synthesis of l-tartrate from l-ascorbate remains limited to a very few species, amongst which we must be grateful to count the domesticated grapevine *Vitis vinifera* and its relatives on which wine production is based. Pathways for the degradation of ascorbate were first proposed ~50 years ago and have formed the basis of more recent biochemical and molecular analyses. The present review seeks to summarize some of these findings and to propose opportunities for future research.

## Teaser

Ascorbate is an important plant metabolite. Its breakdown leads to products including oxalate and tartrate. We review progress towards understanding these pathways and propose further research directions for their elaboration.

## Introduction

A sage comment by Smirnoff (2018) notes that ‘Ascorbic acid … is a simultaneously well-known and surprisingly poorly understood compound’. The limited progress made towards understanding how and why ascorbate degrades *in planta* would at first glance seem to reflect this.

Ascorbate degradation in plants is herein defined as its irreversible catabolism to yield new compounds, in contrast to the reversible oxidation of ascorbate to the monodehydroascorbate radical (MDHA) and dehydroascorbic acid (DHA) and its regeneration via enzymatic reduction, which is critical to cellular redox processes. We do not agree with authors who suggest that these new compounds act as stores of excess cellular ascorbate and we are unaware of any published evidence to support the reversibility of the degradative pathways.

Early studies of the pathways proposed for the synthesis of ascorbate were also the stimulus for work directed to identifying its breakdown products. Notably, between the 1960s and 1980s, Frank Loewus and colleagues in the USA and Kasumi Saito and his team in Japan conducted radiolabelling studies in various plant tissues. Identification, using autoradiography, of the products formed led to the proposal of the pathways that remain the basis of our understanding today (reviewed in Loewus, 1999).

**Fig. 1.**
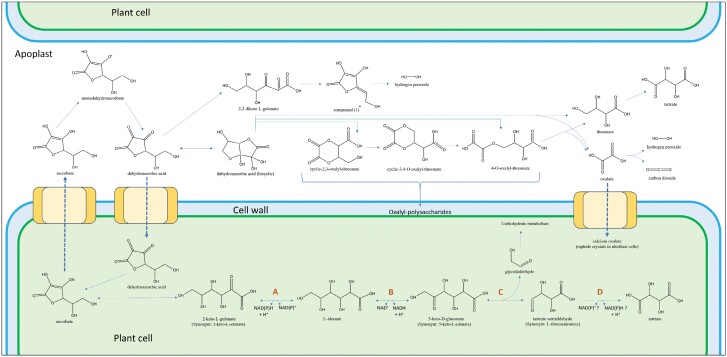
The apoplastic and symplastic pathways of l-ascorbate degradation in plants. Dehydroascorbic acid in the apoplast is formed from the enzymatic oxidation of ascorbate and disproportionation (dismutation) of two molecules of the resulting monodehydroascorbate. Candidate enzymes are indicated by A and B, where A is 2-keto-l-gulonate reductase and B is l-idonate dehydrogenase. Putative activities are indicated by C and D, where C is transketolase and D is tartaric semialdehyde dehydrogenase.

Their work identified two routes of ascorbate breakdown. The first results in the formation of oxalate (C_2_) and threonate (C_4_) via the cleavage of ascorbate (C_6_) between carbons 2 and 3; in the second, tartrate (synonym l-threarate) (C_4_) and glycolaldehyde (C_2_) are formed following the cleavage of ascorbate between carbons 4 and 5 (Fig. 1). In both cases, there is little to no evidence for the further metabolism of many ascorbate breakdown compounds. Rather, the products accumulate either with no proven physiological roles (for instance, tartrate levels can reach several milligrams per gram of fresh weight in ripe grape berries, critically important for the use of grapes in winemaking, but of no evident selective advantage) or, as is the case for oxalate, may play vital roles in sequestering cellular calcium, the softening of cell walls, or in forming prickly crystals of calcium oxalate that may act to deter insect feeding. Oxalyltransferase, a plant cell wall acyltransferase activity, transfers oxalate groups from ascorbate metabolites to carbohydrates, a rare example of the further metabolism of oxalate (Dewhirst and Fry, 2018a). The fate of threonate formed in the 2,3-cleavage pathway remains largely unknown; in some plants, it is further oxidized to form tartrate, which accumulates in leaves. In grape berries, tartroyl esters of many simple phenolic compounds are known to accumulate during ripening, but only to levels of <200 mg l^–1^ (compared with >5 g l^–1^ tartrate) (Waterhouse *et al.*, 2017).

Early work to identify the products of ascorbate degradation was comprehensively reviewed by Loewus (1999) and DeBolt *et al.* (2007); only the briefest of these details will be repeated here. This review will provide an update to earlier work covering both pathways of ascorbate degradation and highlight some of the outstanding questions that remain unanswered.

## Ascorbate breakdown between carbon atoms 2 and 3: the formation of oxalate and l-threonate

It was during the search for the link between ascorbate and tartrate in plants that a pathway for oxalate formation from the breakdown of ascorbate was first proposed (see Loewus, 1999). Extensive studies using a wide range of radiolabelled compounds confirmed that in many plants, carbon atoms 1 and 2 of ascorbate were released, forming oxalate. Further work identified the 4-carbon fragment of the ascorbate as threonate. In plants belonging to the *Geraniaceae*, a 4-electron oxidation was proposed to result in the formation of tartrate. None of the enzymes responsible for these reactions was identified. Subsequent studies (Loewus, 1999; DeBolt *et al.*, 2004) suggested that in grapevines, a minor proportion of ascorbate degradation occurred via such 2,3-cleavage reactions, releasing oxalate residues.

A series of papers, beginning with Green and Fry (2005), presented *inter alia* a model for the degradation of ascorbate via 2,3 cleavage and evidence for the likely cellular location of these activities in the apoplast (the fluid that permeates plant cell walls). This consideration is important in the overall picture of ascorbate metabolism: unlike most compounds, ascorbate is found both symplastically in the cytosol and within organelles, and extraprotoplasmically in the apoplast. In all locations, functional roles have been proposed for its occurrence.

Apoplastic DHA appears to be at a metabolic branch point, with flux from its degradation proceeding via two options, both irreversible: (i) oxidation to threonate plus oxalate or (ii) hydrolysis to 2,3-diketo-l-gulonate (DKG) (Parsons and Fry, 2012). Radiolabelling evidence for both these pathways operating simultaneously in the apoplast *in vivo* in heavy-metal-stressed cells was provided (Farhat and Fry, 2023).

**Fig. 2.**
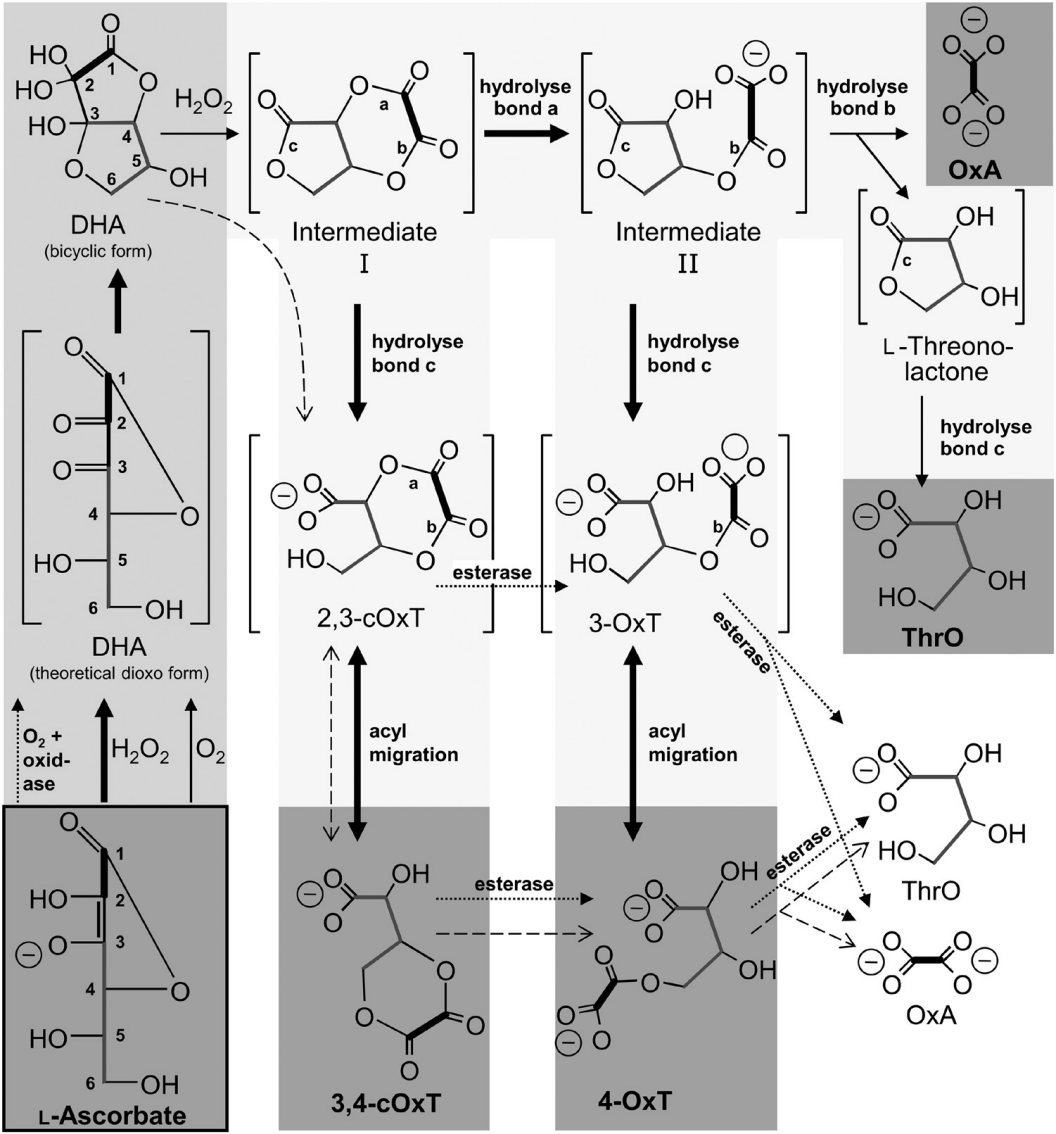
Proposed pathways of ascorbate catabolism. Starting material and major end-products, dark grey background; known oxidation steps, mid-grey background; proposed intermediates, pale grey background. Ascorbate (bottom left) is rapidly oxidized to DHA, which is shown in its conventional dioxo form (centre left) and as the hemiketal monohydrate that predominates in aqueous solution (top left). The C_2_ fragment derived from carbons 1 and 2 of ascorbate is indicated by a bold C–C bond. The C_4_ fragment derived from carbons 3–6 of ascorbate is indicated by dark grey bonds. Hypothetical intermediates are in square brackets. Thick and thin solid arrows show non-enzymatic reactions proposed to be fast and slow, respectively (relative to the preceding step); dotted arrows show reactions catalysed by apoplastic enzymes; dashed arrows show the linear pathway proposed by Green and Fry (2005). No attempt is made to represent stereochemistry except in the two Fischer projection formulae (ascorbate and ‘dioxo’ DHA). Abbreviations not used elsewhere: cOxT, cyclic oxalyl threonate; OxA, oxalate; OxT, oxalyl threonate; ThrO, threonate. Reproduced from Parsons *et al.* (2011) with permission from Portland Press.

Pathway (i) of 2,3-cleavage of ascorbate proposed by Green and Fry (2005) was later proposed to begin with DHA in its bicyclic form (Fig. 2). A series of reactions were characterized, and their products definitively identified following separation by high-voltage paper electrophoresis. All the reactions can proceed non-enzymatically and, in many cases, also enzymatically, leading to the formation of cyclic and non-cyclic oxalyl esters of threonate simultaneously with free oxalate (cyclic:non-cyclic:free ~1:6:1 molar ratio). Subsequent hydrolysis of the esters can lead to formation of additional free oxalate and threonate. The apoplastic nature of these reactions presents the opportunity for the oxalyl esters thus formed to participate in transacylation reactions leading to formation of oxalyl-polysaccharides in the cell wall, which may have a signalling and/or architectural function (Dewhirst and Fry, 2018a), as well as potentially explaining the basis of oxalate formation leading to the synthesis of calcium oxalate raphide crystals in idioblast cells (Green and Fry, 2005).

Pathway (ii) involves the hydrolysis of DHA (a lactone) to form DKG (an open-chain carboxylic acid), which itself is also metabolically labile. Under physiologically relevant apoplast-mimicking conditions, DKG isomerizes (i.e. proceeds by a non-oxidative reaction) via ‘benzylic acid rearrangements’ (probably after re-folding into a new lactone form) to form an interconvertible set of three C_6_ branched-chain compounds: one a free dicarboxylic acid [2-carboxy-l-pentonate] and the other two an epimeric pair of lactones [2-carboxy-l-lyxonolactone and 2-carboxy-l-xylonolactone] each with a single ionizable carboxylate group (Dewhirst *et al.*, 2020). These branched-chain products are themselves unstable, tending to slowly lose one of the two carboxy groups to form the C_5_ sugar-acids l-lyxonate and l-xylonate and their lactones. DKG can also be oxidatively decarboxylated [e.g. by H_2_O_2_, ·OH radicals, and singlet oxygen (^1^O_2_)], forming a new product, 2-oxo-l-*threo*-pentonate (OTP; ‘2-keto-l-xylonate’).

There is thus a large array of products formed, ultimately from ascorbate, via DHA and DKG. The biological ‘roles’ of most of these downstream products are far from clear. Nevertheless, it is of interest that different reactive oxygen species (ROS) tend to generate a different consortium of intermediates and end-products, which may prove a valuable tool in helping researchers to trace which ROS are at work in the apoplast *in vivo* in different physiological situations (Dewhirst and Fry, 2018b). For example, superoxide (unlike H_2_O_2_) yielded negligible OTP; and prolonged H_2_O_2_ treatment oxidatively decarboxylated OTP to free threonate. There is also the intriguing possibility that certain ascorbate catabolites serve as signalling molecules, ‘informing’ the plant of the prevailing redox conditions in its apoplast. It is hoped that this speculation will be explored in the future.

The DHA formed through MDHA disproportionation or ascorbate oxidase activity may also be ‘lost’ to further redox cycling via hydrolysis to DKG. This is irreversible under cellular conditions; the oxidation of DKG may subsequently lead to the formation of H_2_O_2_ and the hydroxyl radical ·OH within the apoplast (Kärkönen *et al.*, 2017).

## Ascorbate breakdown between carbon atoms 4 and 5: the formation of l-tartrate

The 4,5-cleavage pathway leading to the synthesis of tartrate was recently reviewed in detail (Burbidge *et al.*, 2021) and will be described here only briefly. Unlike the enzymatic/non-enzymatic 2,3-cleavage pathway, which has wide botanical distribution, relatively few plant species accumulate tartrate and fewer still do so to any significant level. Low concentrations of tartrate have been reported in many plants, but significant levels are found only in berries of many members of the grapevine family *Vitaceae* and some species of tamarind. This pathway is generally thought to occur in the cytosol, with the resulting tartrate transported to the vacuole where it accumulates to several milligrams per gram of fresh weight.

The pioneering work of Saito and Kasai (1969, 1982, 1984) established the role of l-ascorbate as precursor for the synthesis of tartrate, and—via a series of detailed radiolabelling studies—the sequence of intermediates was identified and confirmed (Malipiero *et al*., 1987). Subsequent biochemical and molecular analyses identified two candidate enzymes (l-idonate 5-dehydrogenase and 2-keto-l-gulonate reductase) for specific stages in this sequence (DeBolt *et al.*, 2006; Jia *et al.*, 2019). 2-Keto-l-gulonate reductase is suggested to catalyse the formation of l-idonate from 2-keto-l-gulonate, whilst l-idonate 5-dehydrogenase is proposed to catalyse the formation of 5-keto-d-gluconate from l-idonate.

There has been only limited success in identifying the mechanisms and enzymes involved in the remaining steps of this pathway. It remains to be determined whether cytosolic ascorbate in its lactonized form is simply hydrolysed, breaking the intramolecular ester linkage between carbons 1 and 4 of ascorbate and, from this open-chain form, a tautomeric rearrangement results in the formation of 2-keto-l-gulonate, or if cytosolic ascorbate is oxidized to DHA and, from this, 2-keto-l-gulonate is formed (which would require a reducing agent). It should be noted that no mechanism has been proposed for this latter reaction. Rather, in the absence of reduction to ascorbate through the activity of DHA reductase, the further hydrolysis of DHA leads to the formation of 2,3-diketo-l-gulonate. No mechanism has been identified to form 2-keto-l-gulonate from 2,3-diketo-l-gulonate.

Similar conjecture surrounds the steps necessary for the last stages of the pathway following the formation of 5-keto-d-gluconate (synonym 5-keto-l-idonate) through the action of l-idonate dehydrogenase. Early work by Saito *et al.* (1997) proposed a hydrolase activity for the reaction cleaving between carbons 4 and 5 of 5-keto-d-gluconate; a subsequent study suggested that transketolase could instead be involved (Salusjärvi *et al.*, 2004). Transketolases are involved in the non-oxidative pentose phosphate pathway (PPP) and in the regeneration of ribulose 5-phosphate in the Calvin cycle. In both cases, donor and acceptor substrates are phosphorylated; for a transketolase to be involved with tartaric acid synthesis, there is a requirement for at least one non-phosphorylated substrate to be recognized. A recent study provides support for this, through the identification of a mutant *Escherichia coli* transketolase with activity against 5-keto-d-gluconate as the donor substrate and acetaldehyde as the acceptor (Wang *et al*., 2022). Su *et al*. (2023) report the occurrence of elevated levels of transcription of one of the isoforms of transketolase, VvTk2, in berries of grapevines with naturally high levels of tartrate. Transient expression in berries resulted in higher levels of tartrate than in controls. Intriguingly, sequence analysis suggested that the enzyme is localized to the chloroplast.

The product of a transketolase-mediated cleavage of 5-keto-d-gluconate between carbons 4 and 5 would be the 4-carbon compound tartaric acid semialdehyde (synonym l-*threo*-tetruronate). A simple two-electron oxidation by a semialdehyde dehydrogenase activity would then be required to form tartaric acid. Further biochemical work is needed to confirm the presence of enzymes in grapevines and other tartrate-accumulating plants capable of carrying out these reactions.

## Discussion and future work

An earlier review of this topic (DeBolt *et al.*, 2007) posed a question of the benefits and costs of irreversibly breaking down ascorbate versus its regeneration for use in cellular redox regulation. It remains to be determined what proportion of ascorbate made in the plant is ‘lost’ to the synthesis of oxalate and threonate, or tartrate, and whether at any time or under any stress-related conditions this presents a problem for the normal functioning of the plant.

Whilst detailed information is known about the identity of most intermediates formed in both pathways, biochemical and molecular data remain sparse. The 2,3-cleavage pathway appears to be mainly apoplastic, beginning with DHA produced in the extraprotoplasmic fluid within the cell wall. The resulting accumulation of soluble oxalate in the apoplast fits with its role in cell wall loosening, associated with developmental processes including fruit ripening. However, oxalate also accumulates as insoluble crystals of calcium oxalate, which may adopt specific shapes including needle-like raphides. In these cases, research has indicated specialized idioblast cell types as the location of oxalate synthesis. Confirmation that the proposed pathway of ascorbate degradation leading to oxalate formation is present within crystal idioblast cells was provided in the work of Kostman *et al.* (2001).

The interesting questions that remain to be asked are focused as much on the physiological ‘why’ as on the molecular/biochemical/chemical ‘how’. Ascorbate has multiple vital roles in ‘dynamic’ plant metabolism and yet, under certain conditions in some plant species, a proportion is removed from redox ‘circulation’ for use in either one or other form of biosynthesis. This is comparable with a common motif in plant specialized metabolism, in which compounds arising in primary metabolism, for instance acetyl-CoA, malate, and erythrose 4-phosphate, form the basis of more complex structures including carotenoids, phenolics, etc. In the case of the degradation of ascorbate, the comparatively simple end-products oxalate, threonate, and tartrate are major outcomes of these pathways. Aside from redox-based modifications, there is little or no further elaboration of the initial precursor compound. Thus, it may be appropriate to consider these reactions part of the plant’s intermediary metabolism, distinguishing them from those involved in primary (energetic/growth) and specialized metabolism pathways.

Oxalate plays important roles in the sequestration of calcium in plant cells, which can include its removal from further chemical and osmotic impact and, in some cases, the provision of protection from herbivory via the accumulation of crystal structures that deter insect attack. Thus, its formation from ascorbate, available either as a translocated compound or one synthesized within a particular cell type, can be readily rationalized.

There is as yet no comparable explanation for the functions of tartrate or threonate, tartrate being found at millimolar levels in grape. It is suggested that tartrate accumulation in the vacuole of immature (green) berries may offer protection against predation by seed dispersal agents (birds) before the seeds are mature and ready for distribution (Ford, 2012). As ripening (of both seed and pericarp) proceeds, the accumulation of sugar and the change of skin colour to dark reds via anthocyanin synthesis and accumulation serve, respectively, as the reward for dispersal and an indicator that the berry is ready for dispersal (i.e. that the seeds within are mature). Alternatively, the accumulation of tartrate has been proposed to fulfil an osmotic function, whereby the expansion of the ripening berry is enabled through water uptake and softening of the cell walls (reviewed in Burbidge *et al.*, 2021). In both cases, for tartrate to be unambiguously linked to these functions, further work with grapevine mutants is required, and the role of the often higher concentrations of malic acid that accumulate in the developing berry must be explained.

It is of interest to reflect that the occurrence of tartrate in grapes is implicitly linked with their use for winemaking, and yet the levels of its synthesis cannot be controlled by any of the cultural or management practices employed in the vineyard to manipulate berry ripeness or the composition of other metabolites. A detailed examination of berry acidity over two contrasting seasons, together with molecular analysis of gene expression in both l-ascorbate and tartrate synthesis pathways, revealed that whilst ascorbate synthesis showed some response to differences in seasonal conditions, tartrate levels and the levels of the gene encoding l-idonate dehydrogenase were minimally affected (Cholet *et al.*, 2016).

The observation of DeBolt *et al.* (2004) that in grape berries radiolabel can be recovered in oxalate and tartrate following feeding with l-[1-^14^C]ascorbate provides evidence for cleavage of ascorbate between carbon atoms 2 and 3 or 4 and 5. These experiments need to be repeated, potentially using stable isotope labelling and MS/MS analysis for quantification of product formation. Other reports of the occurrence of oxalate during grape berry development (Webb *et al.*, 1995; Arnott and Webb, 2000); Hardie *et al.*, 1996; Melino *et al.*, 2009; Simson and DeBolt, 2015), particularly when focused on the formation of crystalline forms of calcium oxalate, for which ascorbate is widely considered as the source of oxalate (Franceschi and Nagata, 2005), support the hypothesis that in the grapevine at least, ascorbate can serve two distinct degradative fates. Of interest in this context are the data of Storey *et al*. (2003), who reported the occurrence of crystals of calcium oxalate in grapevine root tissues. In contrast to leaves and fruit, tartrate does not accumulate in root tissues.

The kinetic data for the two enzymes associated with the pathway of tartrate synthesis (Table 1) are in line with enzymes associated with ‘specialized’ rather than primary metabolism in plants (Bar-Even *et al.*, 2011) In both cases, the *K*_m_ values suggest that intermediates in the pathway accumulate at significant levels during synthesis. This has not been observed *in vivo*, although the early research with radiolabelled precursors and intermediates provided clear evidence for their occurrence (Saito and Kasai, 1982; Malipiero *et al.*, 1987). High-resolution MS/MS analysis is suggested to be an important tool here for the differentiation of intermediates of very similar masses.

**Table 1.** Kinetic data of the enzymes associated with tartaric acid synthesis in grapevines

Enzyme	Cofactor	*V*_max_ (nkatals mg^–1^)	*K*_cat_ (s^–1^)	*K*_m_ (mM)	*K*_cat_/*K*_m_ (s^–1^ M^–1^)
l-Idonate dehydrogenase	NAD^+^	188	7.59	2.2	3.345 × 10^3^
2-Keto l-gulonate reductase	NADH	17.190	9.520	1.561	6.099 × 10^3^
2-Keto l-gulonate reductase	NADPH	7.544	4.178	0.700	5.97 × 10^3^

For l-idonate dehydrogenase, the reaction catalysed was l-idonate+NAD^+^→ 5-keto-d-gluconate+NADH; for 2-keto-l-gulonate reductase, the reactions were 2-keto-l-gulonate+NADH or NADPH→l-idonate+NAD^+^ or NADP^+^

In microbial systems, ascorbate catabolism has been associated with enzymatic steps involving unusual chemistry, ultimately leading to its use as a sole carbon source (Stack *et al.*, 2020). Future work should explore the potential for comparable activities for the degradation of ascorbate in plants.

The diversity of metabolic roles shown by ascorbate is not limited solely to its antioxidant properties and, as shown here, extends into the various products formed from its breakdown. Moreover, as more roles for ascorbate are elucidated in plants, it is likely that its degradation will continue to feature importantly. An example of this was reported by Gerna *et al.* (2021) who made mutant plants of *Arabidopsis thaliana* lacking a functional gene *AtFADH1a* encoding the enzyme fumarylacetoacetate hydrolase (FAH). Seeds of mutant plants, which were longer lived and showed an elevated capacity for germination at 25 °C, contained higher concentrations of DHA, threonate, and ascorbate compared with the wild type. Whilst no suggestion for the role of FAH in ascorbate catabolism was proposed, the observations suggested a redox-regulating function associated with the late-stage development of seeds in *A. thaliana*. Further research will elucidate the chemical and biochemical details of these pathways, and highlight the mechanisms behind their regulation at genetic, developmental, and environmental levels.
